# 584. Beyond the Surface: Clinical and Social Determinants of Pavement Burns

**DOI:** 10.1093/jbcr/irag033.357

**Published:** 2026-04-06

**Authors:** Alexander Chen, Brenna Chen, Vidhani Goel, Alexander Rowan, Christopher Demme, Tiffany Yu, Paul P Kim, Kendra Coleman, Kavita Batra, Stephanie Martinez, Rabia Nizamani

**Affiliations:** University of Nevada Las Vegas, Las Vegas, NV; Leon S. Peters Burn Center, Las Vegas, NV; Kirk Kerkorian School of Medicine at University of Nevada, Las Vegas, NV; Leon S. Peters Burn Center, Las Vegas, NV; Leon S. Peters Burn Center, Las Vegas, NV; Leon S. Peters Burn Center, Las Vegas, NV; Leon S. Peters Burn Center, Las Vegas, NV; Leon S. Peters Burn Center, Las Vegas, NV; Kirk Kerkorian School of Medicine at University of Nevada, Henderson, NV; University of Nevada Las Vegas, Las Vegas, NV; UMC Lions Burn Care Center, Las Vegas, NV

## Abstract

**Introduction:**

With extreme heat in urbanized desert cities, pavement-type contact burns are a growing source of morbidity and mortality. Despite this, limited data characterize the clinical outcomes of pavement-type contact burns compared to other contact burns. This study aims to describe the epidemiology, clinical course, and outcomes between the two groups, focusing on mortality and healthcare resource utilization.

**Methods:**

A retrospective review was conducted of adults admitted with contact burns to a regional burn center between January 1, 2014 and December 31, 2023. Extracted variables included demographics, injury characteristics, social determinants, healthcare utilization, and outcomes. Comparisons between pavement and other contact burns were performed using t-tests, chi-square, or Fisher’s exact tests, with significance set at *p*<.05.

**Results:**

Of 727 patients, 508 (69.9%) had pavement burns. Pavement burns occurred more often in summer (76.2%, *p*<.001) and affected older patients (52.8 ± 19.2 vs. 34.3 ± 26.6 years, *p*<.001), with greater male predominance (71.7% vs. 62.6%, *p*=.015) and higher rates of homelessness (19.8% vs. 1.6%, *p*<.001). Pavement burn patients had larger TBSA (5.8 ± 5.8% vs. 2.5 ± 3.4%, *p*<.001), lower admission GCS (13.1 ± 4.0 vs. 14.8 ± 1.5, *p*<.001), and higher initial temperatures (37.1 ± 2.0 vs. 36.8 ± 1.0°C, *p*=.015). Mortality was significantly higher (7.3% vs. 1.8%, *p*=.003). Length of stay was longer (20.3 ± 29.4 vs. 8.1 ± 10.3 days, *p*<.001), ICU admission was more frequent (48.1% vs. 21.8%, *p*<.001), ICU stays were longer (11.3 ± 18.3 vs. 5.0 ± 6.3 days, *p*=.003), and ventilator use greater (17.9% vs. 2.3%, *p*<.001). Pavement burn patients also had higher Distress Scores (60.3 ± 28.3 vs. 53.1 ± 30.0, *p*=.003) and were more often from at-risk or distressed communities (61% vs. 49%).

**Conclusions:**

Pavement burns represent a disproportionately severe form of contact injury, with higher mortality, increased resource utilization, and greater association with social vulnerability. These findings highlight the urgent need for targeted prevention, seasonal resource planning, and interventions addressing the unique challenges faced by socially and medically vulnerable populations in desert urban environments.

**Applicability of Research to Practice:**

These findings support targeted prevention strategies such as summer-season outreach. They also underscore the need for resource allocation in burn centers during seasonal surges. Protocols for early surgical consultation and ICU triage may improve outcomes.

**Funding for the study:**

N/A.

